# Gastric ectopic pancreatic ductal adenocarcinoma coexisted with endometriosis mimick advanced disease: a case report and literature review

**DOI:** 10.3389/fonc.2025.1573622

**Published:** 2025-07-24

**Authors:** Jingwen Wang, Xia Zhu, Fei Liu, Jiayan Chen, Xi Tang

**Affiliations:** ^1^ Department of Oncology, Huadong Hospital, Fudan University, Shanghai, China; ^2^ Department of Pathology, Huadong Hospital, Fudan University, Shanghai, China

**Keywords:** ectopic pancreas, stomach, malignancy, immunotherapy, chemotherapy

## Abstract

Ectopic pancreas (EP) is most commonly located in the upper gastrointestinal tract, particularly in the stomach. Malignant transformation of EP is exceptionally rare, and due to its very low incidence, the response to drug therapy and overall prognosis remain poorly understood. This report presents a case of gastric ectopic pancreatic ductal adenocarcinoma. Unusually, the patient also had concurrent endometriosis, which caused the tumor to resemble advanced cancer on imaging. Although over 50 cases of malignant transformation of EP have been documented. Nevertheless, it has not been definitively established which drug therapeutic regimens is effective for this disease. This is the first to detail the tumor’s response to anti-tumor drug therapy. Pancreatic cancer is generally resistant to anti-tumor drugs. However, in this case, the tumor arising from EP regressed following treatment with immune checkpoint inhibitors (ICIs) combined with chemotherapy. This observation highlights differences in the biological behavior of these two tumor types.

## Introduction

Ectopic pancreas (EP) is defined as isolated pancreatic tissue that develops outside the pancreas and lacks both anatomical and vascular connections to the orthotopic pancreas. It is considered a congenital abnormality associated with abnormal embryonic development (1). The most widely accepted hypothesis regarding its pathogenesis is the misplacement theory, which suggests that pancreatic progenitor cells are ectopically deposited into the developing gastrointestinal tract during foregut rotation (2, 3). The stomach (35%) is the most common location of EP tissue, followed by the duodenum (34%) and proximal jejunum (13%) (4). On endoscopy, EP typically appears as a subepithelial lesion, making it difficult to differentiate from other subepithelial tumors (5, 6). Endoscopic ultrasonography is useful for improving diagnosis due to its ability to detect ductal structures and cystic components characteristic of EP (7). Endoscopic ultrasound-guided fine needle aspiration/biopsy is often necessary to reach the diagnosis of EP, especially when malignant transformation is suspected (8).

However, malignant transformation of EP is extremely rare. Due to the absence of specific clinical manifestations, most cases are diagnosed incidentally through surgery or biopsy. To date, more than 50 well-documented cases of malignant transformation of EP have been reported (3). Surgical excision is a recommended treatment for malignant EP. The available evidences fail to establish which specific antitumor agents demonstrate efficacy in this subset of patients, nor does it clarify whether they may derive clinical benefit from immune checkpoint inhibitors. This report presents a case of adenocarcinoma arising from EP in the stomach. This case is particularly notable because the patient also had concurrent endometriosis. Since endometriosis showed increased 18F fluorodeoxyglucose (18F FDG) uptake on positron emission tomography CT (PET CT), it created a false impression of advanced-stage cancer. However, to the best of our knowledge, this is the first report describing successful treatment of this condition with immune checkpoint inhibitors (ICIs) combined with chemotherapy.

## Case description

**Figure 1:**
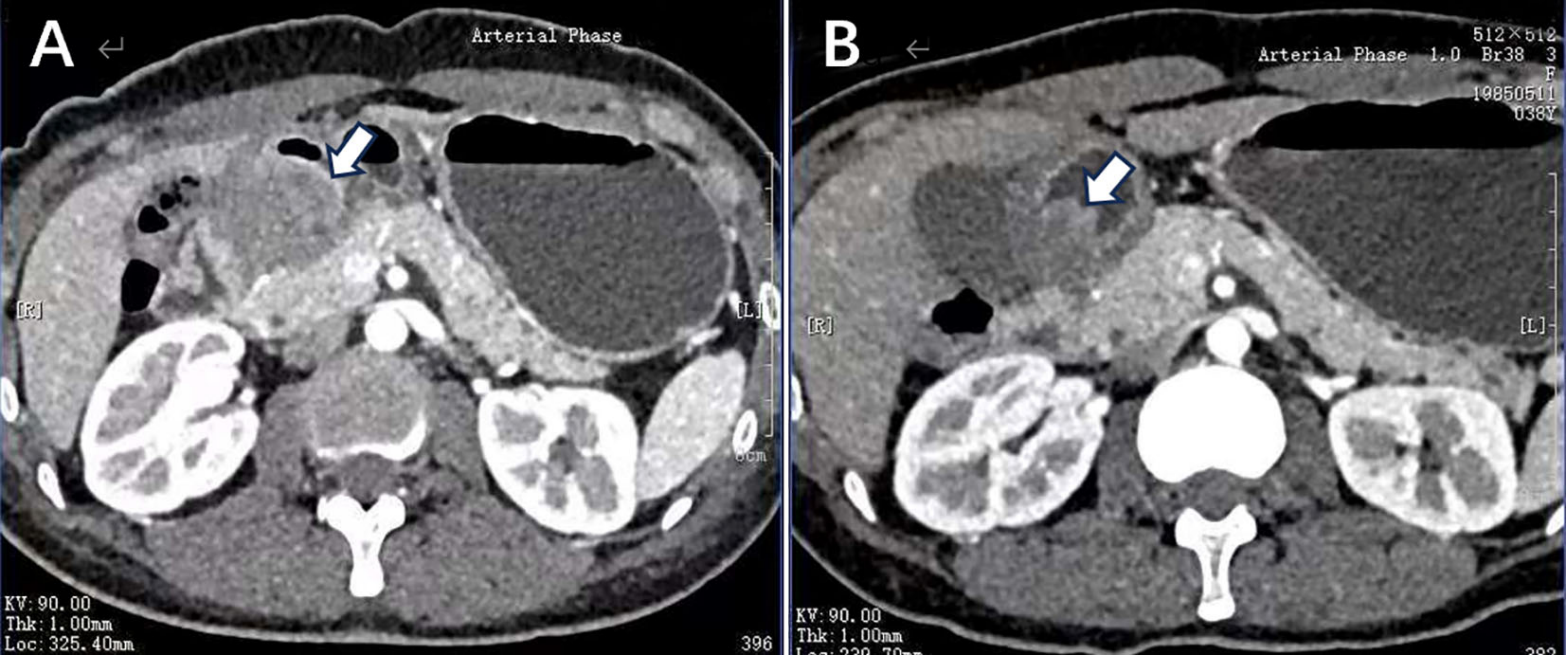
**(A)** Before systemic therapy, enhanced CT image showed edema of the gastric antrum, with a lesion located between the gastric antrum and pancreatic head demonstrating heterogeneous enhancement. **(B)** After the treatment of nivolumab plus XELOX, the follow-up imaging revealed reduced edema in the gastric antrum and a decrease in the size of lesion.

**Figure 2:**
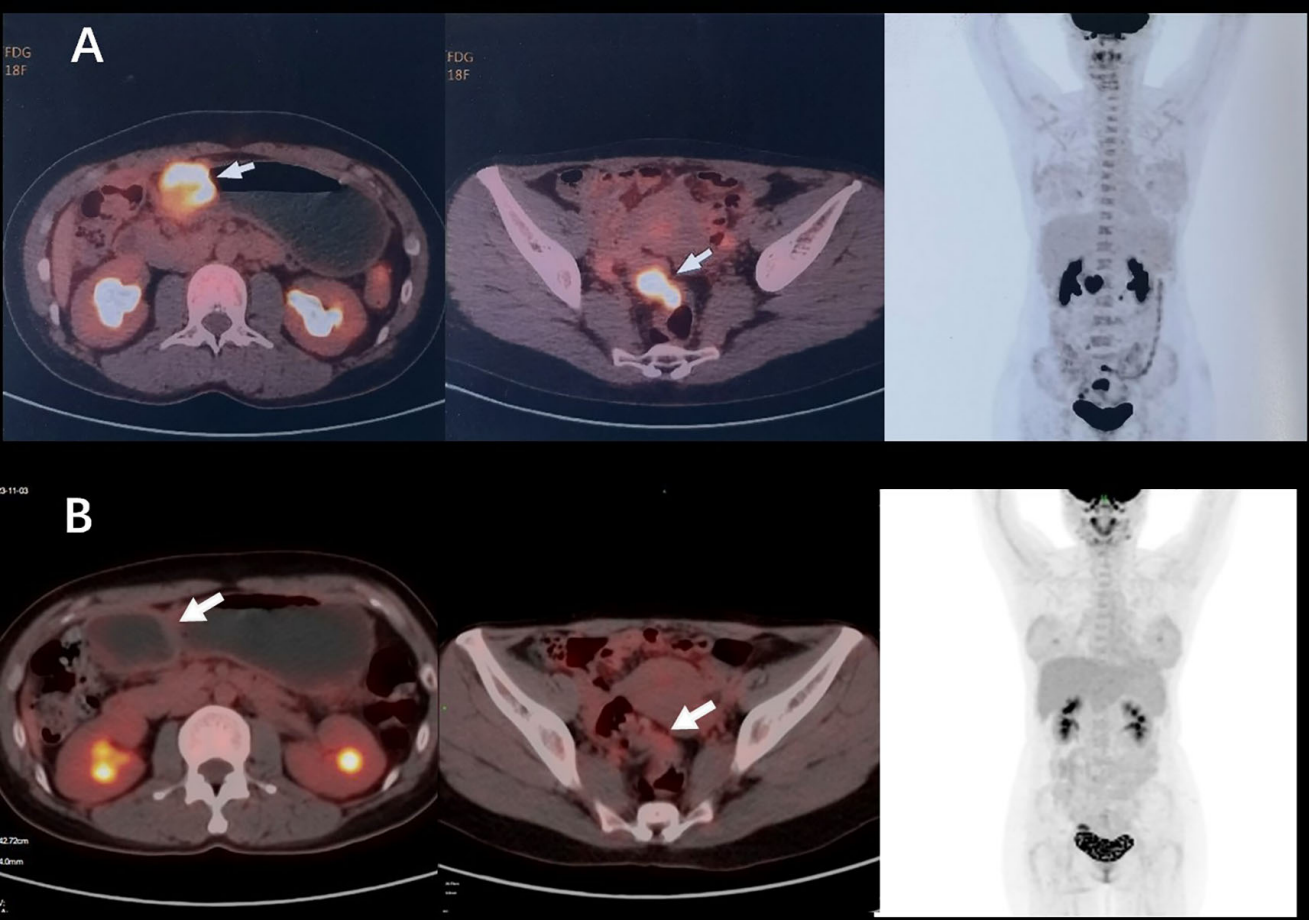
**(A)** Before systemic therapy, PET-CT showed multiple suspicious malignant lesions located at gastric antrum (SUVmax 8.0), rectosigmoid junction (SUVmax 9.4), and peritoneum without hypermetabolic activity. **(B)** After the treatment of nivolumab plus XELOX, PET-CT showed a residual lesion only at the rectosigmoid junction (SUVmax 3.1), and no hypermetabolic activity in the gastric region.

**Figure 3:**
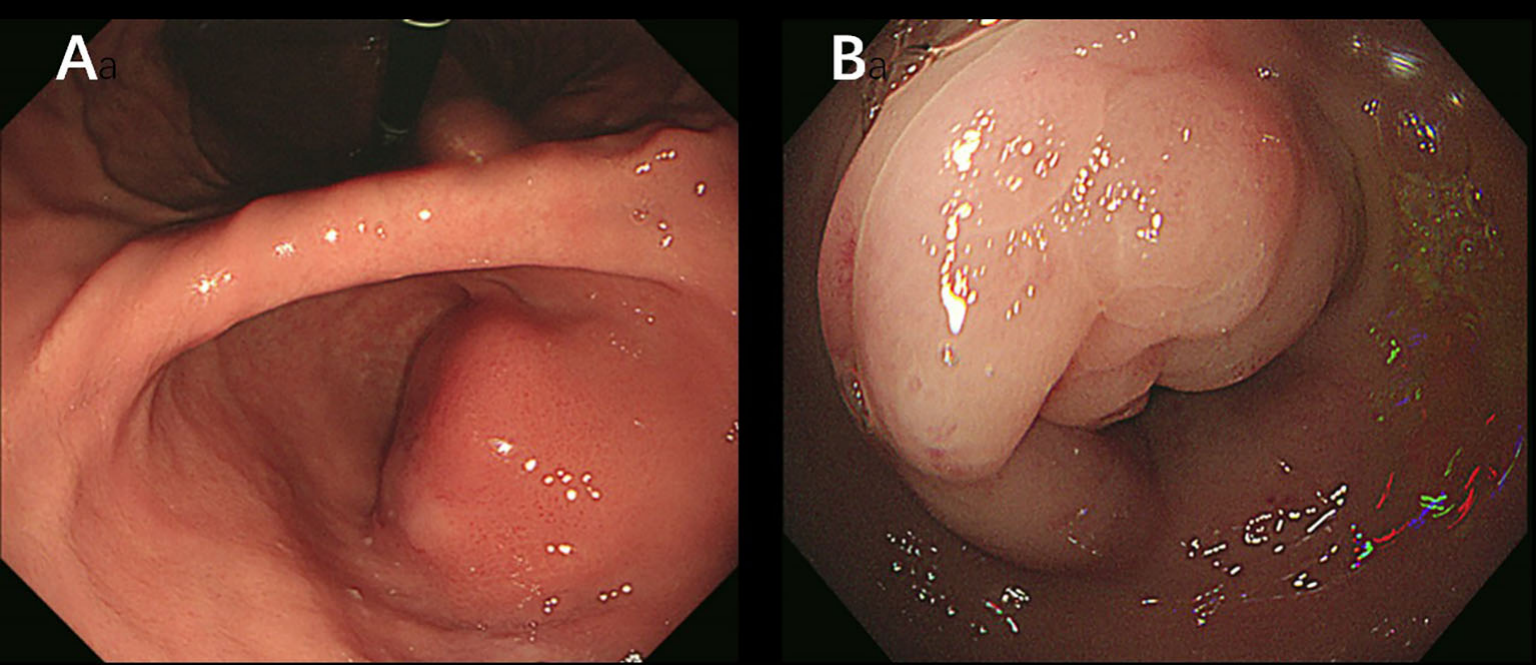
**(A)** Upper gastrointestinal endoscopy image shows a subepithelial lesion in the gastric antrum. **(B)** Colonoscopy image shows a subepithelial lesion at the rectosigmoid junction.

A 38-year-old woman was admitted to the hospital in August 2023 with a 1-month history of right lower abdominal pain. The patient denied a history of chronic and genetic diseases. Physical examination revealed that the abdomen was soft with mild tenderness in right lower abdominal. Abdominal computed tomography (CT) revealed thickening of the greater omentum and two lesions in the gastrointestinal tract: one between the gastric antrum and pancreatic head and another at the rectosigmoid junction (**Figure 1A**). Abnormally elevated concentrations of multiple serum tumor markers, especially CA199, were observed (**Table 1**). Positron emission tomography-computed tomography (PET-CT) showed: (i) a suspected malignant lesion in the gastric antrum with increased 18F fluorodeoxyglucose (18F FDG) uptake (SUVmax 8.0); (ii) increased 18F FDG uptake at the rectosigmoid junction (SUVmax 9.4), suggesting either implantation metastasis or a second primary tumor; and (iii) thickening of the greater omentum and mesentery without hypermetabolic activity (**Figure 2A**). Upper gastrointestinal endoscopy revealed a subepithelial lesion in the gastric antrum (**Figure 3A**), while colonoscopy identified a subepithelial lesion at the rectosigmoid junction (**Figure 3B**). However, endoscopic biopsies from both sites did not detect malignant cells. To establish a pathological diagnosis, transabdominal puncture of the gastric mass was performed under ultrasound guidance. Pathological examination revealed moderately differentiated adenocarcinoma in the gastric tissue. Immunohistochemistry (IHC) showed tumor programmed death ligand 1 (PD-L1) expression with a combined positive score (CPS) of approximately 10 (**Table 1**). Human epidermal growth factor receptor 2 (HER2) protein was positive (++) (**Table 1**), but HER2 gene amplification was negative as assessed by fluorescence *in situ* hybridization (FISH) (data not shown). Based on these findings, the patient was diagnosed with advanced gastric adenocarcinoma. Between August 2023 and October 2023, she received three cycles of nivolumab (360 mg every 3 weeks) combined with XELOX chemotherapy (capecitabine 1000 mg/m² twice daily on days 1–14, and oxaliplatin 130 mg/m² on day 1, every 3 weeks). In November 2023, follow-up CT and PET-CT scans indicated partial remission, with a residual lesion only at the rectosigmoid junction (SUVmax 3.1), and no hypermetabolic activity in the gastric region (**Figures 1B**, **2B**). Serum CA199 levels gradually returned to normal (**Table 1**). Given the significant reduction in tumor burden and the patient’s strong preference for surgical treatment, laparoscopic abdominal exploration was performed in November 2023. Intraoperative findings showed a smooth omentum without metastatic lesions. The patient underwent distal subtotal gastrectomy with D2 lymphadenectomy, resection of the colon lesion, and total hysterectomy.

**Table 1:** Pre/post-therapeutic parameters of laboratory and imaging examinations.

Parameters of laboratory/image	Pre-neoadjuvant therapy	Post-neoadjuvant therapy
CA199	41.3U/mL	12.5U/mL
CA125	66.9 U/mL	1.26U/mL
CA153	28.4 U/mL	21.4 U/mL
SUVmax on PET-CT	gastric antrum:8.0 rectosigmoid junction:9.4	gastric antrum:3.1 rectosigmoid junction:0
PD-L1 CPS in tumor issue	10	2
HER2 IHC in tumor issue	++	+

**Figure 4:**
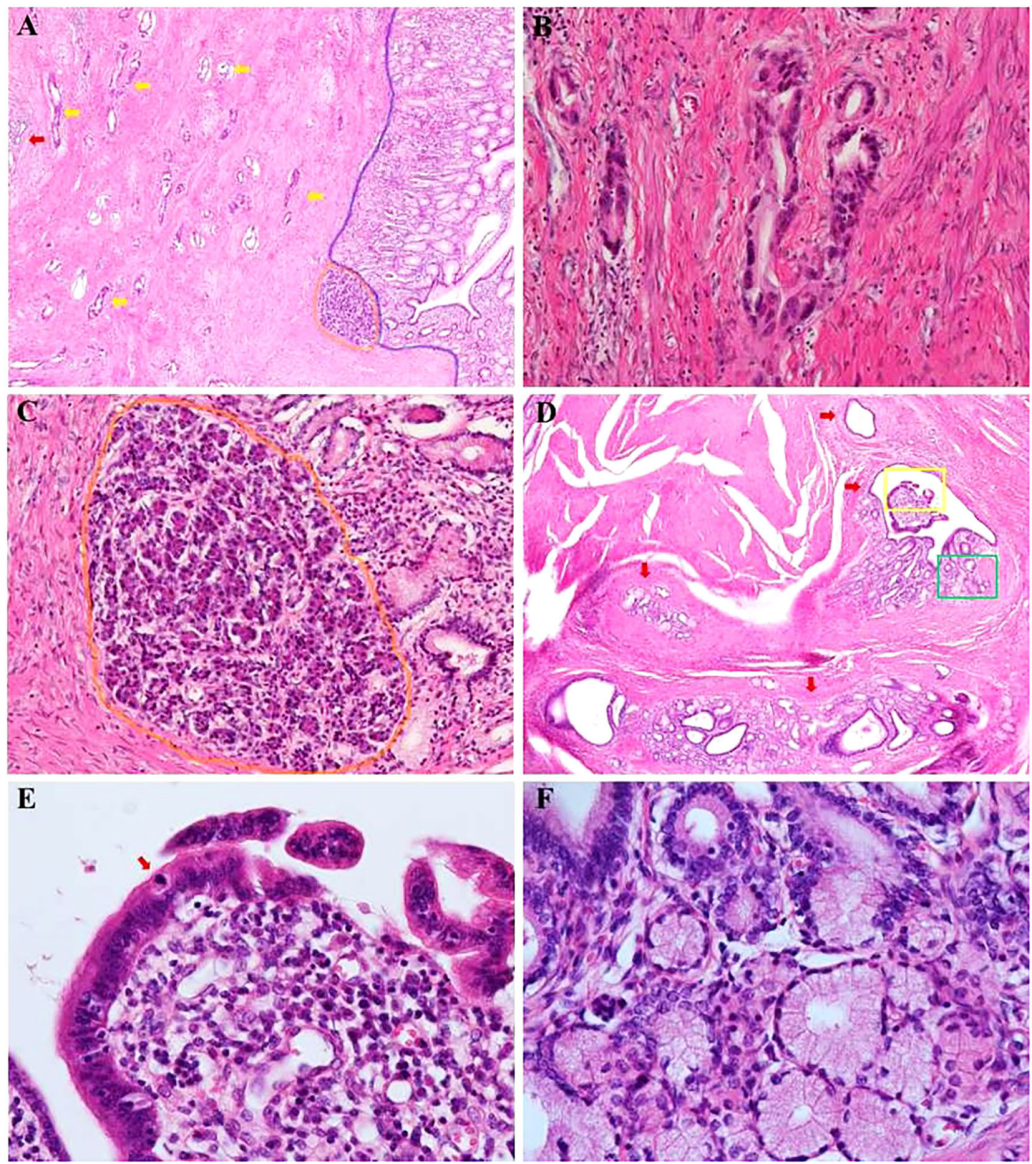
Pathologic findings of major distal gastrectomy. **(A)** Hematoxylin and eosin–stained sections showing chronic non-atrophic gastritis changes on the surface gastric mucosa (the right area of blue line, ×40). Ectopic pancreatic acini are deep in the mucosa near the muscular layer of the mucosa (area within the orange circle, ×40). Moderately differentiated tubular adenocarcinoma infiltrating the intrinsic muscular layer with post-treatment changes (shown by the yellow arrow, ×40), with EP duct next to them (shown by the red arrow, ×40). **(B)** Magnification of the adenocarcinoma area (×200). **(C)** Magnification of the EP follicles area (×200). **(D)** EP ducts are seen in other areas. Within the walls of the larger ducts, there are lobular structures formed by aggregates of smaller ducts. In some area, low-grade pancreatic intraepithelial neoplasia (PanIN) is identified in the EP ductal epithelium (region indicated by red arrow, ×100). **(E)** High magnification (×400) in the yellow box of **(D)** showing enlarged nuclei with moderate atypia and overlapping nuclei in the low-grade PanIN region. A mitotic figure is visible (the area indicated by red arrow). **(F)** High magnification (×400) in the green box of **(D)** showing low-grade PanIN with simple, columnar, mucin-filled, perfectly polarized cells.

Pathological histology revealed chronic non-atrophic gastritis changes on the surface of the gastric mucosa. EP acini were observed deep within the mucosa, near the muscularis mucosae (**Figures 4A, C**). Moderately differentiated tubular adenocarcinoma infiltrated the muscularis propria, with evidence of post-treatment changes (**Figures 4A, B**). In some areas, low-grade pancreatic intraepithelial neoplasia (PanIN) was identified in the EP ductal epithelium (**Figures 4D-F**). No lymph node metastasis was detected. The presence of normal gastric mucosa on the surface and dysplasia within the EP supported the diagnosis of malignancy originating from EP, classified as Heinrich type III.

**Figure 5:**
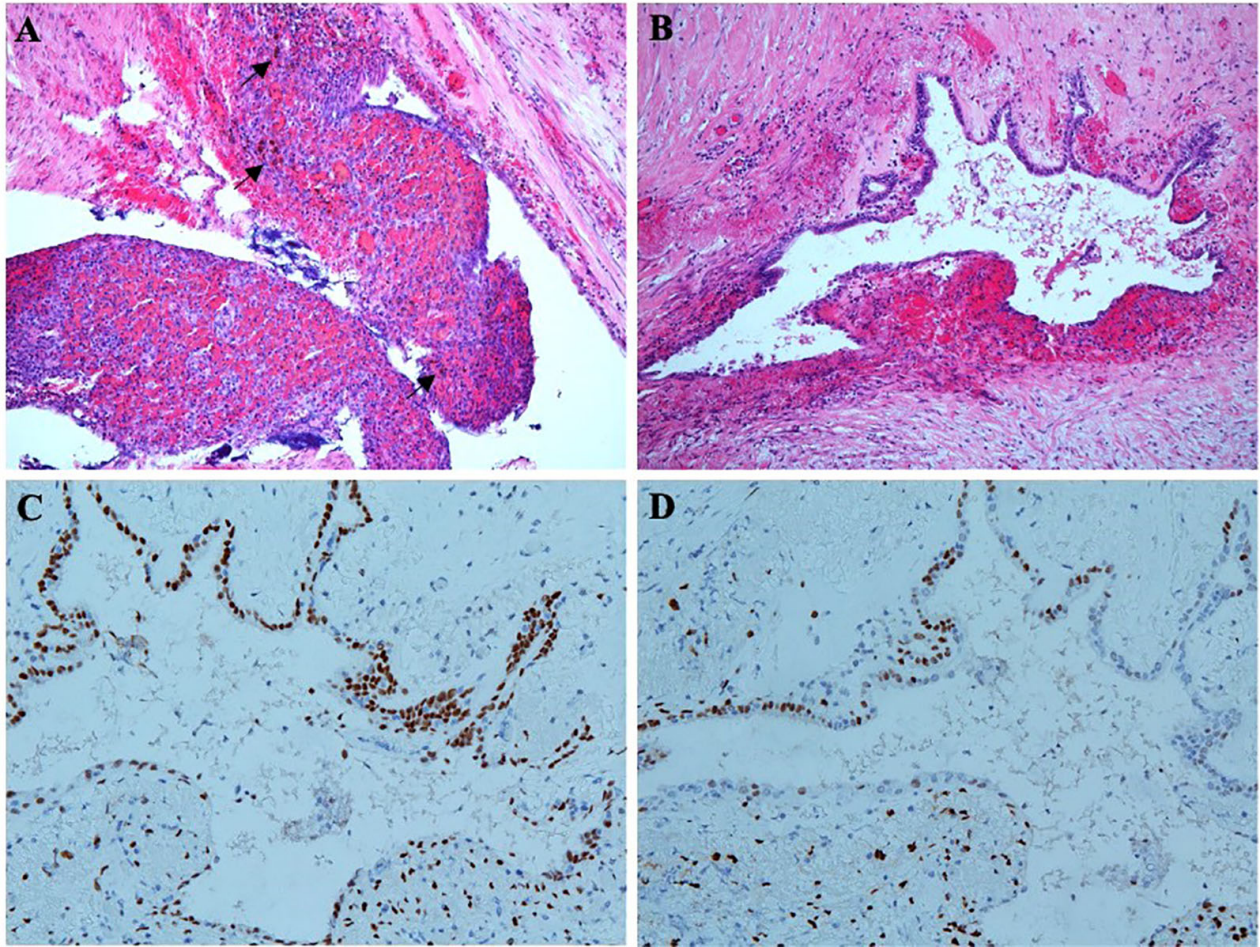
Pathologic findings of radical rectal resection. **(A)** Ectopic endometrial glands and distinct endometrial mesenchyme are on the serosal surface of the rectal wall. Ferrous haemoflavin deposits are observed in focal areas (shown by black arrows). **(B)** Ectopic endothelial glands are also located in the intrinsic muscular layer of the intestinal wall, accompanied by hemorrhage. **(C)** Immunohistochemistry shows ectopic endometrial glands and stroma expressing ER. **(D)** Immunohistochemistry shows ectopic endometrial glands and stroma expressing PR.

Unexpectedly, microscopic examination of the rectal lesion revealed endometriosis. The rectal mucosa appeared normal, but ectopic endometrial glands and stromal tissue were observed on the serosal surface of the rectal wall (**Figure 5A**). Ferrous haemoflavin deposits were noted in focal areas (**Figure 5A**), indicating previous hemorrhage. Ectopic endometrial glands were also identified within the muscularis propria of the intestinal wall, accompanied by interstitial hemorrhage (**Figure 5B**). Immunohistochemistry confirmed that the ectopic endometrial glands and stroma expressed estrogen receptor (ER) (**Figure 5C**) and progesterone receptor (PR) (**Figure 5D**). All 11 perirectal lymph nodes showed chronic inflammation.

Based on these pathological findings, the postoperative pathological staging was determined as ypT2N0M0. The patient received three cycles of adjuvant nivolumab combined with XELOX, followed by planned adjuvant nivolumab monotherapy for 11 cycles. The patient tolerated the treatment well without severe adverse effect. During the follow-up period, no tumor recurrence was observed, and the patient remained disease-free for over 12 months.

## Discussion

EP was first histologically identified by Klob in 1859 (2). The incidence of EP, as estimated through autopsy and surgery, ranges from 0.5% to 13.7% (5, 9). Gaspar Fuentes et al. modified Heinrich’s histological classification of EP into four categories: type I, consisting of typical pancreatic tissue with ducts, acini, and islet cells; type II, primarily composed of acini with few ducts and no islet cells; type III, mainly composed of ducts with few or no acini and no islet cells; and type IV, consisting exclusively of endocrine islets (10, 11). According to this classification, our case is type III, characterized by fully developed acini and ductal structures but no islet cells.

Malignant transformation of EP is extremely rare, and its exact incidence is unknown. Jun et al. conducted a systematic pathological study of 165 EP cases, finding acinar-ductal metaplasia in 86.7% of cases and pancreatic intraepithelial neoplasia (PanIN) or intraductal papillary mucinous neoplasms (IPMNs) in 50.4%, but no cases of malignant transformation (4). Cazacu et al. reported that most malignant transformations of EP occur in Heinrich type I lesions, predominantly located in the stomach (3). Various histological types of malignant EP have been reported, with adenocarcinoma being the most common. Other types include neuroendocrine tumors, cystadenocarcinomas, pseudopapillary neoplasms, anaplastic carcinomas, and acinar cell carcinomas (3). In this case, the malignancy was an adenocarcinoma arising in a Heinrich type III EP, which is the least common type reported in the literature (3, 12).

The diagnosis in this case adhered to the rigorous criteria proposed by Guillou et al., which are widely used. The criteria include: (1) malignant tumor cells located within or adjacent to EP tissue; (2) evidence of a transitional zone between pancreatic elements and carcinoma, while excluding invasion by metastatic or adjacent malignant tumors; and (3) non-neoplastic EP tissue containing fully developed acinar and/or ductal structures (13). In this case, a continuous progression from PanIN to adenocarcinoma was observed in the EP tissue, supporting the diagnosis. Additionally, elevated serum CA199 levels corroborated that the tumor originated from pancreatic tissue.

This case is remarkable due to the coexistence of two types of heterotopias: EP and endometriosis. To the best of our knowledge, this is the first reported case of adenocarcinoma arising from EP coexisting with endometriosis and mimicking advanced malignancy. Endometriosis is a common condition, with a prevalence of 6% to 10% in fertile women. In addition to hormonal factors, inflammatory mediators are involved in its pathogenesis (14). Inflammation-induced glucose metabolism may explain why endometriosis can be visualized on 18F-fluorodeoxyglucose positron emission tomography/computed tomography (18F-FDG PET/CT). Balogova et al. reported that 18F-FDG PET/CT detected 55% of endometriosis sites, with maximum standardized uptake values (SUVmax) ranging from 1.8 to 5.3 (median, 3.8) (15). Mild 18F-FDG uptake can potentially mimic malignant lesions. Wang et al. described a case where 18F-FDG uptake in endometriosis mimicked a primary pelvic malignancy with lymph node metastasis (16). Similar reports (17, 18) have shown mildly elevated serum CA125 levels in these patients, a finding consistent with our case. In this patient, metabolically active lesions regressed following anti-tumor therapy. However, due to a lack of systematic studies, it remains uncertain whether this change in uptake was influenced by hormonal factors or chemotherapy.

Pancreatic cancer is generally considered “immune-privileged” due to a paucity of infiltrating T cells and the abundance of immunosuppressive cells, such as regulatory T cells (Tregs) and myeloid-derived suppressor cells (MDSCs), within its tumor microenvironment (19). Consequently, agents targeting the PD-1/PD-L1 checkpoint on T cells have shown limited efficacy in pancreatic cancer (20). While chemotherapy is the standard first-line treatment, its impact on improving prognosis is limited. For instance, the NAPOLI-3 trial demonstrated that the combination of fluorouracil, leucovorin, liposomal irinotecan, and oxaliplatin (NALIRIFOX) extended median overall survival (OS) by only 1.9 months compared to albumin-bound paclitaxel with gemcitabine (21). Even with first-line chemotherapy, the median OS for pancreatic cancer rarely exceeds 12 months. Due to its rarity, no standard systemic therapy regimen exists for malignant EP. Considering its pancreatic origin, it is reasonable to assume that malignant EP might exhibit resistance to both chemotherapy and immunotherapy.

In this case, EP was not identified before surgery, and the tumor was treated as a common gastric adenocarcinoma. The CheckMate 649 trial demonstrated that nivolumab combined with XELOX (capecitabine and oxaliplatin) significantly improved OS, PFS, and objective response rate compared to XELOX alone in patients with metastatic, non-HER2-positive gastric cancer, especially those with PD-L1 combined positive scores (CPS) ≥5 (22). Other studies, such as GEMSTONE-303, Orient-16, KEYNOTE-859, have also demonstrated a positive correlation between CPS levels and survival time in these patients undergoing immunochemotherapy (23–25). Consistently, CPS of 5 is the most commonly established cut-off value. Given the tumor’s PD-L1 CPS of approximately 10, nivolumab combined with XELOX was selected for treatment. The patient responded well to this regimen, enabling surgical intervention. Given the ultra-low incidence of malignant tumors of EP, whether its tumor microenvironment characteristics align with pancreatic tumors remain unknown. Interestingly, reports suggest that malignant EP has a better prognosis than primary pancreatic cancer (3). Our case manifests a pronounced efficacy to immunochemotherapy, suggesting a less aggressive tumor biology and more intra-tumoral lymphocytic infiltration. This may indicate differences in the biological behavior of the two tumor types, potentially influencing their response to immunotherapy.

To the best of our knowledge, this is the first reported case of malignant transformation of EP coexisting with endometriosis and mimicking advanced gastric cancer. While radical tumor resection remains the most effective treatment, a combination of ICIs and chemotherapy may also improve prognosis.

## Data Availability

The raw data supporting the conclusions of this article will be made available by the authors, without undue reservation.
